# Meier-Gorlin syndrome

**DOI:** 10.1186/s13023-015-0322-x

**Published:** 2015-09-17

**Authors:** Sonja A. de Munnik, Elisabeth H. Hoefsloot, Jolt Roukema, Jeroen Schoots, Nine VAM Knoers, Han G. Brunner, Andrew P. Jackson, Ernie MHF Bongers

**Affiliations:** Department of Human Genetics 836, Institute for Genetic and Metabolic Disease, Radboud University Medical Center, PO Box 9101, 6500 HB Nijmegen, The Netherlands; Department of Clinical Genetics, Erasmus Medical Center, Rotterdam, The Netherlands; Department of Pediatrics, Division of Respiratory Medicine, Radboud University Medical Center, Amalia Children’s Hospital, Nijmegen, The Netherlands; Department of Medical Genetics, Center for Molecular Medicine, University Medical Center Utrecht, Utrecht, The Netherlands; Medical Research Council Human Genetics Unit, Institute of Genetics and Molecular Medicine, Western General Hospital, Edinburgh, UK

**Keywords:** Meier-Gorlin syndrome, Ear patella short stature syndrome, Microtia, Short stature, Patellar a-/hypoplasia, Pre-replication complex, Origin recognition complex

## Abstract

Meier-Gorlin syndrome (MGS) is a rare autosomal recessive primordial dwarfism disorder, characterized by microtia, patellar applasia/hypoplasia, and a proportionate short stature. Associated clinical features encompass feeding problems, congenital pulmonary emphysema, mammary hypoplasia in females and urogenital anomalies, such as cryptorchidism and hypoplastic labia minora and majora. Typical facial characteristics during childhood comprise a small mouth with full lips and micro-retrognathia. During ageing, a narrow, convex nose becomes more prominent. The diagnosis MGS should be considered in patients with at least two of the three features of the clinical triad of microtia, patellar anomalies, and pre- and postnatal growth retardation. In patients with short stature and/or microtia, the patellae should be assessed with care by ultrasonography before age 6 or radiography thereafter. Mutations in one of five genes (*ORC1*, *ORC4*, *ORC6*, *CDT1*, and *CDC6*) of the pre-replication complex, involved in DNA-replication, are detected in approximately 67-78 % of patients with MGS. Patients with *ORC1* and *ORC4* mutations appear to have the most severe short stature and microcephaly.

Management should be directed towards in-depth investigation, treatment and prevention of associated problems, such as growth retardation, feeding problems, hearing loss, luxating patellae, knee pain, gonarthrosis, and possible pulmonary complications due to congenital pulmonary emphysema with or without broncho- or laryngomalacia. Growth hormone treatment is ineffective in most patients with MGS, but may be effective in patients in whom growth continues to decrease after the first year of life (usually growth velocity normalizes after the first year) and with low levels of IGF1. At present, few data is available about reproduction of females with MGS, but the risk of premature labor might be increased.

Here, we propose experience-based guidelines for the regular care and treatment of MGS patients.

**Electronic supplementary material:**

The online version of this article (doi:10.1186/s13023-015-0322-x) contains supplementary material, which is available to authorized users.

## Review

In this review, we outline the clinical symptoms of MGS and propose experience-based guidelines for care and treatment of MGS patients. The reported percentages of clinical symptoms are calculated from the frequency of these symptoms in the 38 patients with bi-allelic mutations in one of the five causative pre-replication complex genes (*ORC1*, *ORC4*, *ORC6*, *CDT1*, and *CDC6*) described in literature [1–5]. Clinical symptoms that may affect management and that are present only in patients with clinically diagnosed MGS (in whom the molecular defect is unknown) are mentioned as well.

### Disease name and synonyms

Meier-Gorlin syndrome (MGS; MIM#224690, ORPHA 2554).

Ear patella short stature syndrome (EPS).

### Definition

Meier-Gorlin syndrome (MGS) is characterized by the triad of microtia, absent or small patellae and short stature. At least two of these three clinical features are present in 97 % (32/33) of patients with MGS, the combination of patellar a-/hypoplasia and microtia being the most prevalent. Microtia, however, can be mild. One patient was described with short stature, but without microtia or patellar anomalies, indicating that the clinical phenotype might be more variable than previously suspected [3, 4].

### Epidemiology

Until now, 67 patients with MGS have been described since the first reported patients by Meier in 1959 and Gorlin in 1975 (30 males; 36 females; 1 of unknown sex; age range 0.3-55 years) [1–22]. The underlying molecular defect is known in 38 of these patients [1–5].

The exact prevalence of MGS has not been determined, but is estimated to be less than 1-9/1,000,000 based on the number of cases described in literature. However, this might be an underestimation, due to underreporting and missed diagnoses.

### Clinical description

Patients with MGS present with a recognizable phenotype. The classical triad of clinical features comprises microtia, patellar aplasia or hypoplasia, and pre- and postnatal growth retardation.

Microtia is present in almost all patients with MGS (34/36; 94 %). Severity ranges from mild to severe microtia, where the ears appear underdeveloped and low-set. Narrow ear canals and conductive hearing loss may accompany microtia.

Patellar anomalies are among the most frequent findings in MGS (31/33; 94 %). In most patients, patellae are absent, but they may be hypoplastic.

Prenatal growth is delayed in the majority of patients with MGS. Intra uterine growth retardation (IUGR) was present in 97 % (35/36), with a mean birth weight of −3.8 SD (range < −6.5 SD to −0.3 SD), five infants were born prematurely (5/30; 17 %; terms ranging from 28 to 36 weeks of gestation) [2–4]. In three cases, the pregnancy was terminated because of severe IUGR, in combination with congenital anomalies [5].

We studied growth extensively in our cohort of 45 MGS patients (35 patients with a known and 10 with an unknown molecular defect) [23]. Postnatal growth was delayed during the first year of life. Growth velocity was almost normal thereafter, with patients growing parallel to the normal growth charts without significant catch up growth. In this cohort, height varied considerably between MGS patients and ranged from −9.6 SD to −0.4 SD. The average adult height in the MGS patients with a known underlying molecular defect was −5.5 SD (5 patients).

Microcephaly (head circumference < −3SD) was present in 43 % (13/30) of patients, with a head-circumference ranging from −9.8 SD to +1.7 SD. Height differed significantly between the gene mutated, ethnic background and gender. Patients with mutations in *ORC1* and *ORC4* had a significantly shorter stature (a difference of 4,7 SD and 3.1 SD, respectively) and smaller head circumference (a difference of 5.0 SD and 1.6 SD, respectively), than patients with mutations in *ORC6*, *CDT1*, or *CDC6* [4, 23].

Growth hormone therapy was unsuccessful in MGS patients with a known molecular defect. However, growth hormone therapy was successful in two patients with a clinical diagnosis of MGS, whose growth velocity continued to be low after the first year and who had low levels of IGF1 (−3.3 SD and −4.6 SD) [23].

All postpubertal females (10/10; 100 %) had mammary hypoplasia. Transvaginal ultrasound investigations were performed in five adult females (clinically diagnosed with MGS). A small uterus was reported in three of them and polycystic ovaries were reported in two of these five females [23].

Axillary hair is often sparse or absent, while pubic hair generally is normal in both males and females.

**Fig. 1:**
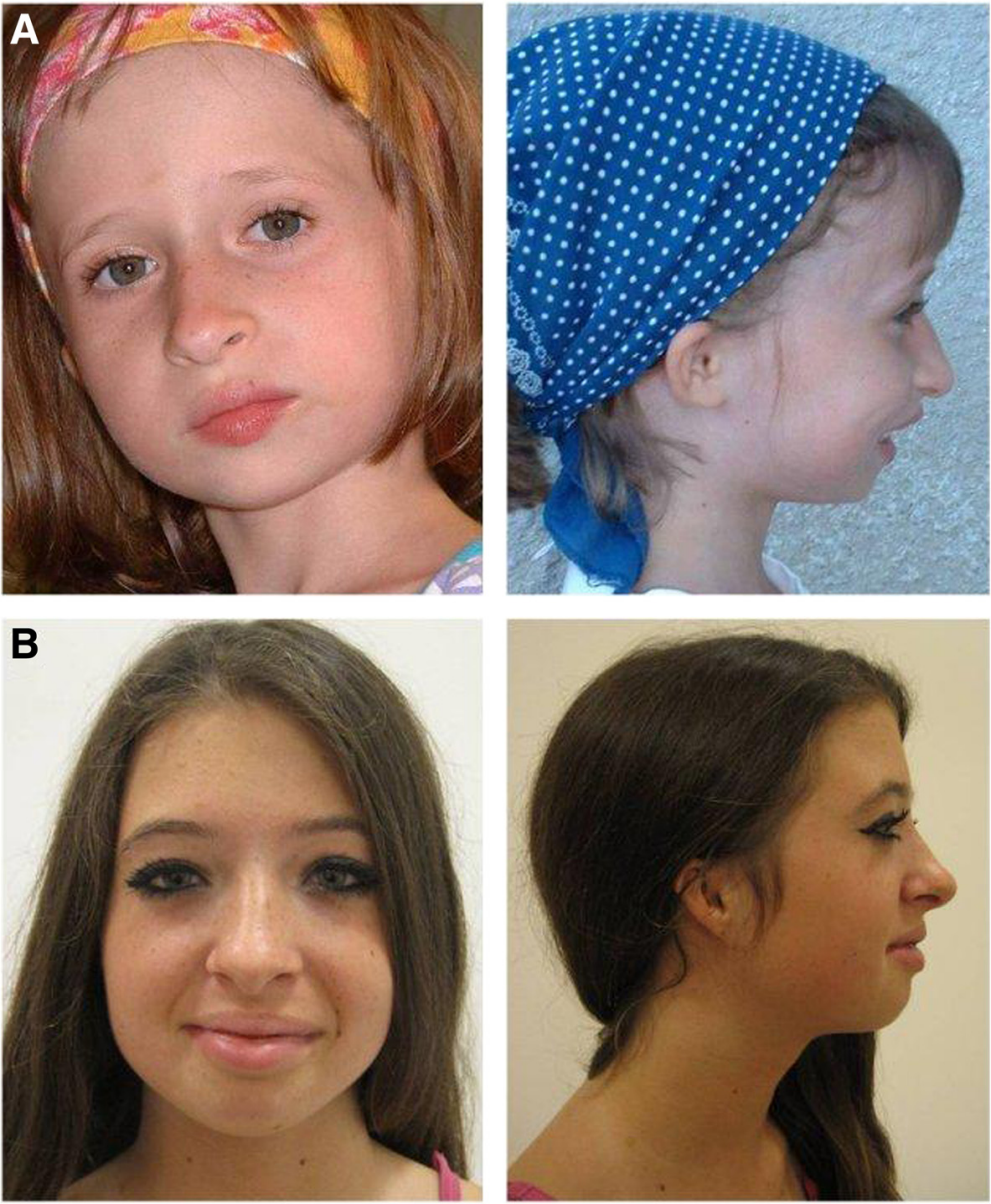
Facial features of Meier-Gorlin syndrome. Patient A is a girl aged 7 years and 9 months with two mutations in *ORC4*. Patient B is a 14 year old girl, who carries one mutation in *ORC1*. Both show the characteristic facial features of MGS, including microtia, a prominent nose with a convex nasal profile, a small mouth with full lips, and retro-/micrognathia

Furthermore, patients with MGS have a recognizable facial phenotype with microstomia, full lips and retro-/micrognathia at young age (Fig. 1). The nose can be narrow and convex with a high nasal bridge. These characteristics of the nose become more prominent with age.

Intellect is normal in the majority of patients with MGS (30/31; 97 %), although delayed motor development and/or speech development without intellectual disability (ID) were present in 19 % (6/32) and 16 % (5/32) of patients, respectively. A mild ID was present in one patient (3 %).

Respiratory tract anomalies are relatively frequent. Congenital pulmonary emphysema was reported in 43 % (12/28) of patients with MGS. Congenital pulmonary emphysema is usually detected in the neonatal period or early in childhood(10/12; 83 %), but can be diagnosed late in childhood. Bronchomalacia, laryngomalacia and/or tracheomalacia are reported in 42 % (10/24) of subjects. Recurrent respiratory tract infections occur often during childhood, but disappear thereafter.

Congenital cardiac anomalies appear to be rare: they have been described in two patients (2/30; 7 %: one ventricular septal defect, one patent ductus arteriosus).

Feeding problems are seen in approximately 80 % (26/32) of patients with MGS during infancy and young childhood. Problems range from a small appetite (which may correlate with the short stature of infants), to gastroesophageal reflux with administration of tube feeding or gastrostomy interventions in 35 % (11;31).

Anomalies of the urogenital tract are frequently reported, especially cryptorchidism in males (9/14; 64 %) and hypoplastic labiae in females (5/21; 24 %).

A delayed bone age and genu recurvatum are often seen in patients with MGS. Joint contractures, including club feet, were present in 23 % (7/30) of patients.

Not much is known about the reproduction of patients with MGS. Recently, a previously described woman clinically diagnosed with MGS gave birth prematurely twice after 17 and 18 weeks of gestation, respectively [23].

### Aetiology

MGS is an autosomal recessive disorder. Mutations in five genes of the pre-replication complex (*ORC1*, *ORC4*, *ORC6*, *CDT1* and *CDC6)*, were detected in patients with MGS. Mutation detection rate was approximately 78 % in a cohort of 45 patients with MGS [4]. The pre-replication complex assembles on genomic DNA at origins of replication.

The exact underlying mechanism for the clinical features of MGS remains to be elucidated. The impaired function of the pre-replication complex (PRC) is presumed to reduce the G1 phase of DNA replication and to limit the available time for origin licensing. This limitation is hypothesized to become rate-limiting, thereby impeding cellular proliferation. This would result in a reduction of the total cell number and thereby diminish overall growth [24, 25]. Another possible mechanism affecting cell cycle progression was identified in ORC1 depleted cells, where a defect in the rate of formation of primary cilia was demonstrated to influence cell cycle progression [26]. Alternatively, the ORC1 subunit of the PRC contains distinctive domains that regulate centriole and centrosome copy number. It is suggested that mutations in these domains alter centrosome duplication and thereby contribute to the growth retardation and microcephaly in MGS [27].

Mutations in *ORC1* and *ORC4* appear to cause a more severe short stature and microcephaly than mutations in other genes. Furthermore, compound heterozygous missense- and loss of function mutations appear to have a more severe effect on the phenotype than homozygous or compound heterozygous missense mutations [4]. No patients are known with homozygous or compound heterozygous loss of function mutations, suggesting that these mutations cause a lethal phenotype. No additional genotype-phenotype correlations were recognized.

### Diagnostic methods

The clinical diagnosis of MGS can be established in the presence of microtia, patellar aplasia or hypoplasia, and proportionate short stature. However, about 18 % (6/33) of patients show only two out of three cardinal features, and one patient was described with short stature only.

The diagnosis MGS should be considered in patients with short stature or microtia, and the presence of these features necessitates comprehensive examination of the patellae. In infants, ultrasound investigations are advised, since patellae are radiolucent in the first 5–6 years of life and will not be visible by conventional radiography.

Furthermore, the characteristic facial features and mammary hypoplasia are specific findings for MGS and will assist in diagnosing MGS.

In a patient clinically suspected to have MGS, the diagnosis can be confirmed by detecting compound heterozygous or homozygous mutations in one of the five pre-replication complex genes (*ORC1*, *ORC4*, *ORC6*, *CDT1*, and *CDC6*). Mutations were detected in approximately 78 % of patients clinically suspect for MGS [4]. In patients with a severe short stature and/or microcephaly, starting with the analysis of *ORC1* and *ORC4* should be considered, since mutations in these two genes are associated with a significantly shorter stature and smaller head circumference than mutations in the other genes [4, 23].

### Differential diagnosis

The association of microtia, patellar anomalies and short stature as such has not been described in other syndromes.

MGS is a part of the primordial dwarfism spectrum. These disorders are characterized by microcephaly and prenatal short stature. Other primordial dwarfism disorders include Seckel syndrome and Microcephalic Osteodysplastic Primordial Dwarfism (MOPD) type I, II and III. However, even though these syndromes show considerable overlap with MGS, they are clinically distinct disorders. In Seckel syndrome, short stature is proportionate, as in MGS. However, height and head circumference are generally much smaller in Seckel syndrome than in MGS (average −7.1SD and −8.7SD, respectively [28]), the facial appearance differs from MGS, and patients generally have ID. These patients can have patellar anomalies and microtia, but these findings are not frequently present, contrary to MGS, where they are mandatory features.

Patients with MOPD type 1 and 2 have a disproportionate short stature, and a skeletal dysplasia, clearly distinguishing these disorder from MGS. MOPD type 3 is rare and it is suggested this is the same disorder as MOPD type 1. Stature in patients with MOPD type 3 was proportionate, though.

Patellar anomalies and short stature are cardinal features of RAPADILINO and genitopatellar syndrome. However, these syndromes differ from MGS in the lack of microtia (both), the presence of radial ray defects (RAPADILINO), and developmental delay and agenesis of the corpus callosum (genitopatellar syndrome).

If patellar anomalies are present, and microtia and short stature are absent, nail patella syndrome should be considered. In nail patella syndrome, familial patellar a-/hypoplasia is almost always accompanied by nail dysplasia, including the pathognomic tri-angular lunulae. An overview of other syndromes with patellar anomalies, such as small patella syndrome, patella aplasia-hypoplasia, and trisomy 8 mosaicism is provided by Bongers et al [29]. In patients with microtia, other diagnoses to contemplate are branchio otorenal syndrome, Townes-Brocks syndrome, Treacher-Collins syndrome, Nager syndrome, Miller syndrome, and CHARGE association.

### Genetic counselling

MGS is an autosomal recessive disorder. The recurrence risk for a couple with an affected child is 25 %. Parents are obligate heterozygotes. Heterozygotes are asymptomatic. In literature, three patients with a classic MGS phenotype and mono-allelic mutations (one in *ORC1*, two in *CDT1*) were described. However, their healthy fathers carried the same mutation. Therefore, it is expected that these patients either carry a second mutation in *ORC1* or *CDT1* that cannot be detected with the current molecular techniques, or that their symptoms were caused by a different molecular defect.

To our knowledge, so far, no patients with MGS have had any children. However, future reports are expected. Offspring of a patient with MGS are obligate heterozygotes. The risk of having an affected child with MGS is low (<1 %), since carrier frequencies and thus the risk of a partner being a carrier are low.

### Antenatal diagnosis

When the underlying molecular defect is known, prenatal diagnosis by chorionic villus sampling or amniocentesis is possible. If no causative gene defects are identified, prenatal ultrasound investigations at 18–20 weeks of gestation may contribute to the recognition of MGS. However, it is unknown whether overall growth is already delayed at this term. Microtia and congenital pulmonary emphysema can be seen and certain facial features, such as micrognathia may be detected. Nonetheless, because of the variable expression of the disorder and the lack of structural congenital anomalies, ultrasound abnormalities may be absent, or mild and difficult to interpret. The ethical issues of prenatal diagnosis and possible termination of a pregnancy for a disorder with a variable expression, and a relatively low chance of life-threatening complications or intellectual disability, should be discussed.

### Management

Especially during childhood, multidisciplinary care is needed.

Here, we propose guidelines for regular care and treatment, based on clinical experience. An overview of the proposed guidelines for diagnostic evaluation and management of patients with MGS is presented in Additional file 1: Table S1. 

#### Ears

Microtia can be associated with narrow ear canals and conductive hearing loss. Therefore, at the time of diagnosis, a patient suspected to have MGS should be examined by an ear-nose-throat specialist. Afterwards, examinations are indicated when hearing loss appears to be present.

#### Patellae

Patellar aplasia or hypoplasia may lead to instability of the knee joint, pain and early gonarthrosis. A multidisciplinary approach, including examination by an orthopedic surgeon and rehabilitation physician is advised. Special shoes and advise about strength enhancing exercises and sports, may be provided, especially since patients with MGS often have hypermobile joints and are more prone to develop pes planus.

#### Growth and growth hormone treatment

Growth hormone treatment was effective in only four patients, one with *ORC4* mutations, one with *CDC6* mutations and two without mutations [4, 23]. In one patient with *CDC6* mutations, height improved from −5 to −3 SD during the first 4 years. In two patients without mutations, height improved from −6.8 SD to −3.0 SD and from −5.7 SD tot −3.7 SD.

In a child with MGS, endocrinological evaluation (IGF1 and stimulated growth hormone measurements) is advised. Growth hormone treatment should be considered in a patient with low IGF1 levels and further delay of growth after the first year.

#### Feeding problems

Feeding problems can be severe, necessitating tube feeding and a gastrostomy to optimize nutrition. Gastroesophageal reflux as a cause should be considered.

#### Congenital pulmonary emphysema

Surgical resection can be performed when respiratory distress is life-threatening or debilitating. In older patients with MGS and congenital pulmonary emphysema treatment with selective Beta2-antagonists can be considered.

#### Congenital cardiac anomalies

Since 7 % of the reported MGS patients had a congenital cardiac anomaly, we advise cardiac screening (physical examination, ECG and ultrasound investigations) at the time of diagnosis.

#### Mammary hypoplasia

Treatment of mammary hypoplasia with exogenous estrogen can be debated in females with MGS. Estrogen therapy was reported to be successful in two females with MGS. In a third female, minor effects were reported. In two other females, no effect was seen [4, 23].

Breast augmentation surgery can be considered.

#### Reproduction

Males born with cryptorchidism may have fertility problems. One female with MGS had two consecutive miscarriages. Her uterus was described to be small. No information about other pregnancies is available, but preconceptional gynaecologic examinations should be considered and possible preterm labor should be a point of attention in obstetric care of females with MGS, especially since gynecologic evaluation in four other females with MGS revealed a small uterus and polycystic ovaries in two of them [23].

### Prognosis

Life span is expected to be normal for most individuals with MGS. Osteoarthritis of the knees, secondary to patellar anomalies, may occur at a younger age than usual.

Life threatening complications that may accompany MGS are severe congenital pulmonary emphysema and cortical malformations. When severe congenital anomalies and/or severe respiratory problems are absent, however, there is no reason to expect an abnormal life span.

Four reported patients with MGS were deceased (11 %), and three pregnancies were terminated because of ultrasound abnormalities, including severe IUGR (8 %) [3–5, 16].

One patient (with *ORC1* mutations) passed away after 3,5 months. He had a severe cortical dysplasia, pachygyria and ventricular enlargement, cranial suture stenosis, congenital emphysema of the lung, and his pancreatic tail was absent. His brother died in utero after 17 weeks of gestation. Both patients exhibited microtia and a severe growth retardation. A third patient (with *CDT1* mutations) deceased after a sudden cardiac arrest. He had congenital pulmonary emphysema for which he required surgery. His sister passed away after 3 months. She had severe respiratory problems due to a tracheobronchomalacia with progressive pulmonary emphysema.

### Unresolved questions

The long-term risks of congenital pulmonary emphysema in patients with MGS need to be evaluated through long-term follow-up. Furthermore, future reports about reproduction in patients with MGS have to be awaited to assess the exact reproductive risks.

Approximately 22 % of patients with a classical clinical phenotype of MGS lacked mutations in one of the five known genes of the pre-replication complex [4]. These patients might have mutations in one of the five known genes we are unable to detect with current sequencing techniques, or their symptoms may be caused by mutations in other genes of the pre-replication complex, or in pathways connected to this complex.

It is currently unknown why patients with *ORC1* and *ORC4* mutations appear to have shorter stature and smaller head circumference compared to patients with mutations in other genes. Further understanding of the role of the different genes of the pre-replication complex during growth and development might contribute to gain insight in these differences.

## Conclusions

Here, we provide an overview of the clinical features of Meier-Gorlin syndrome and guidelines for management and treatment of associated problems in this rare disorder. MGS is an autosomal recessive primordial dwarfism disorder characterized by microtia, patellar a-/hypoplasia and short stature and often accompanied by feeding problems, respiratory problems, mammary hypoplasia and urogenital anomalies.

Multidisciplinary care, directed towards diagnosis and treatment of ear anomalies and/or hearing loss, patellar anomalies, feeding problems, respiratory problems, and possible reproductive issues, is necessary to assure optimal medical care. Endocrinologic testing should be routinely performed in all children with MGS, although growth hormone treatment is not beneficial in most patients with MGS.

Molecular analysis of the five known genes for MGS (*ORC1*, *ORC4*, *ORC6*, *CDT1*, and *CDC6*) is important in genetic counseling. A molecular diagnosis assists in determining the exact recurrence risk and enables future reproductive options, such as preimplantation genetic diagnosis and invasive diagnostic testing during pregnancy.

Since a molecular diagnosis cannot be established in 20-35 % of patients with MGS, further studies are necessary to identify the underlying genetic defects in these patients.

## Consent

Written informed consent was obtained from the patients for the publication of this report and any accompanying images.

## Additional file

Additional file 1: Table S1. Proposed guidelines for diagnostic evaluation and management of patients with Meier-Gorlin syndrome based on clinical experience. (DOCX 20 kb)

## Abbreviations

MGS: Meier-Gorlin syndrome
ID: Intellectual disability
ORC1: Origin recognition complex subunit 1
ORC4: Origin recognition complex subunit 4
ORC6: Origin recognition complex subunit 6
CDT1: Chromatin licensing and DNA replication factor 1
CDC6: Cell division cycle 6
MOPD: Microcephalic osteodysplastic primordial dwarfism
