# Supplementary material for: Meier-Gorlin syndrome
Source: Orphanet J Rare Dis. 2015 Sep 17;10:114. doi: 10.1186/s13023-015-0322-x (PMC4574002; doi:10.1186/s13023-015-0322-x)
Supplement: Additional file 1: Table S1. — Proposed guidelines for diagnostic evaluation and management of patients with Meier-Gorlin syndrome based on clinical experience. (DOCX 20 kb) [file 13023_2015_322_MOESM1_ESM.docx]

**Supplementary table 1. Management advice for diagnostic evaluation and management of patients with Meier-Gorlin syndrome based on clinical experience.**

| **Age** | **Organic system** | **Signs and symptoms** | **Investigation** | **Period of evaluation** | **Treatment options** |
| --- | --- | --- | --- | --- | --- |
| **From birth**  **till late childhood** | ***Ears*** | Small external auditory meatus  Conductive hearing loss | Otoscopy  Hearing tests | At diagnosis and annually when indicated | Hearing aids |
|  | ***Skeleton*** | Patellar a-/hypoplasia  Knee complaints, hypermobile joints, pes planus | Knee imaging: ultrasonography ≤ 5-6 y, X-rays > 5-6 y.  Orthopedic assessment | At diagnosis and in case of signs and symptoms | Rehabilitation advice  Arch support  Surgical intervention |
|  | ***Growth*** | Impaired growth velocity after 1 year of age  Low IGF1 | 1. Evaluation of growth 2. Complete endocrine work-up | 1. At diagnosis and annually 2. At diagnosis and in case of signs and symptoms | Growth hormone  treatment |
|  | ***Central nervous system*** | Delayed motor development, muscle weakness  Speech delay | Developmental assessment | Annually | Speech therapy  Extra assistance at school |
|  | ***Gastrointestinal tract*** | Poor weight gain, poor intake  Gastro-esophageal reflux | 1. Monitor feeding and growth 2. Esophageal pH monitoring | 1. Annually 2. In case of signs and symptoms | Nasal tube feeding  GERD treatment^I^ |
|  | ***Respiratory tract*** | Respiratory tract infection  Laryngo-/tracheomalacia  Congenital pulmonary emphysema | 1. Physical examination 2. Evaluation by pulmonary specialist; CAT scan | 1. At diagnosis 2. In case of signs and symptoms | Antibiotics when indicated  Surgery when life-  threatening/ disabilitating  Selective Beta2-agonists  when indicated |
|  | ***Genitourinary tract*** | Cryptorchidism in males  Hypoplastic labiae in females | 1. Physical examination 2. Ultrasound investigations of the inguinal region in males | 1. At diagnosis 2. In case of symptoms and signs | Orchidopexy |
|  | ***Heart*** | Congenital cardiac defect | 1. Physical investigation 2. Cardiac ECG and ultrasound | 1. At diagnosis 2. At diagnosis | Cardiac surgery |
| **From childhood onwards** | ***Skeleton*** | Knee complaints, arthrosis | Orthopedic assessment | In case of signs and symptoms | Surgical intervention |
|  | ***Secondary sexual development*** | Mammary hypoplasia in females  Sparse/absent axillary hair in both males and females | Physical examination | Annually during puberty  In case of signs and symptoms | Estrogen treatment  Breast augmentation surgery |
|  | ***Genitourinary tract*** | Irregular menses, polycystic ovaries, small uterus | Gynecologic assessment^II^ | In case of signs and symptoms | Hormonal treatment when diagnosed with polycystic ovaries^III^ |
|  | ***Pregnancy*** | Possible risk of premature delivery | Antenatal care in a secondary care centre | During pregnancy |  |

^I^ Gastroesophageal reflux disease (GERD) treatment according to the guidelines of the Dutch society of Pediatrics[1]:

- Symptoms of GERD < 18 months of age: conservative treatment by thickening feeds can be considered, but treatment with ranitidine or proton pump inhibitors for 2-4 weeks can be started directly.

Symptoms of GERD > 18 months of age: consider treatment with proton pump inhibitors.

^II^ In case of polycystic ovaries, assessment according to the Rotterdam guidelines[2]:

- Physical examination of the external sexual characteristics and signs of hyperandrogenism
- Ultrasound investigations of the internal sexual organs
- Laboratory assessment to establish androgen excess and/or ovalutory dysfunction.

^III^ Hormonal treatment[3]:

- Consider treatment with hormonal contraceptives if menses occurs less than four times a year.
- In case of a child wish, consider ovulation induction by clomifene citrate.

**References**

1. Benninga MA, Berger MY, Venmans LM, Tabbers M. [Guideline 'Gastroesophageal reflux disease in children from 0-18 years'].

Nederlands tijdschrift voor geneeskunde. 2014;158:A6970.

2. Rotterdam EA-SPcwg. Revised 2003 consensus on diagnostic criteria and long-term health risks related to polycystic ovary syndrome

(PCOS). Hum Reprod. 2004;19(1):41-7.

3. Legro RS, Arslanian SA, Ehrmann DA, Hoeger KM, Murad MH, Pasquali R, et al. Diagnosis and treatment of polycystic ovary syndrome: an Endocrine Society clinical practice guideline. The Journal of clinical endocrinology and metabolism. 2013;98(12):4565-92.
